# Living with brain metastases—A longitudinal qualitative study of patient experiences from time of diagnosis

**DOI:** 10.1093/nop/npag019

**Published:** 2026-03-21

**Authors:** Tonje Lundeby, Asta Bye, Olav Eric Yri, Torunn Elin Wester, Nina Aass, Sjur Bjørnar Hanssen, Stein Kaasa, Marianne Jensen Hjermstad

**Affiliations:** European Palliative Care Research Centre (PRC), Department of Oncology, Oslo University Hospital and Institute of Clinical Medicine, University of Oslo, Oslo, Norway; European Palliative Care Research Centre (PRC), Department of Oncology, Oslo University Hospital and Institute of Clinical Medicine, University of Oslo, Oslo, Norway; Department of Nursing and Health Promotion, Faculty of Health Sciences, OsloMet-Oslo Metropolitan University, Oslo, Norway; European Palliative Care Research Centre (PRC), Department of Oncology, Oslo University Hospital and Institute of Clinical Medicine, University of Oslo, Oslo, Norway; Regional Advisory Unit for Palliative Care, Department of Oncology, Oslo University Hospital, University of Oslo, Oslo, Norway; Regional Advisory Unit for Palliative Care, Department of Oncology, Oslo University Hospital, University of Oslo, Oslo, Norway; European Palliative Care Research Centre (PRC), Department of Oncology, Oslo University Hospital and Institute of Clinical Medicine, University of Oslo, Oslo, Norway; Regional Advisory Unit for Palliative Care, Department of Oncology, Oslo University Hospital, University of Oslo, Oslo, Norway; Regional Advisory Unit for Palliative Care, Department of Oncology, Oslo University Hospital, University of Oslo, Oslo, Norway; European Palliative Care Research Centre (PRC), Department of Oncology, Oslo University Hospital and Institute of Clinical Medicine, University of Oslo, Oslo, Norway; European Palliative Care Research Centre (PRC), Department of Oncology, Oslo University Hospital and Institute of Clinical Medicine, University of Oslo, Oslo, Norway; Regional Advisory Unit for Palliative Care, Department of Oncology, Oslo University Hospital, University of Oslo, Oslo, Norway

**Keywords:** brain metastases, emotional responses, patient-centered care, qualitative study, symptom burden, thematic analysis

## Abstract

**Background:**

Brain metastases (BM) are prevalent intracranial neoplasms in adults, affecting 20%-40% of cancer patients. With improved systemic therapies and neuroimaging, the frequency of BM diagnoses is rising. Despite advancements and longer survival time, the prognosis remains poor, with survival rates ranging from 3 months to over a year, depending on the diagnosis. This qualitative study provides insight into the patients' needs, experiences, and perspectives upon BM diagnosis. The aim of the study was to explore how patients experience being diagnosed with BM, their care and follow-up, and how BM impacts their lives and concerns.

**Methods:**

A qualitative study using longitudinal semi-structured interviews with patients recently diagnosed with first-time BM. Participants were recruited from one Norwegian hospital, with interviews conducted at 3 intervals over 4 months. Inclusion criteria included age ≥18, verified BM diagnosis, and ability to consent and participate in interviews. Inductive thematic analyses were performed to identify overarching themes.

**Results:**

Twenty-two patients participated, with interviews revealing 4 themes: (1) BM as either an additional burden or more of the same, (2) trust in the healthcare system despite unmet needs, (3) distancing from illness, and (4) acceptance of and adjustment to symptom burden. Patients expressed varied emotional responses, practical challenges, and evolving information needs over time.

**Conclusion:**

Patients diagnosed with BM face multifaceted challenges. A patient-centered approach, emphasizing clear communication, symptom management, and tailored care, is essential. Understanding patient experiences can help healthcare providers offer personalized care. Continued research is needed to address the unique needs of this population and improve care practices.

Key Points

- Brain metastases (BM) present significant challenges for patients, despite advances in diagnosis and treatment.
- Patients face practical challenges, evolving symptoms, and changing information needs throughout their care journey.
- A patient-centered approach emphasizing clear communication, tailored care, and symptom management is essential, as patient needs may change over time, making it crucial to monitor and adapt care accordingly.
- Understanding patients' experiences can help healthcare providers improve care and address the unique needs of this population.

Importance of the Study

Brain metastases profoundly affect patients' lives, causing substantial emotional, cognitive, and physical challenges. Despite advances in treatment, there is limited understanding of how patients experience their diagnosis, care, and follow-up. This study underscores the importance of addressing unmet patient needs through the provision of tailored information, proactive symptom management, and involvement in treatment decisions, recognizing that the desire for engagement varies widely. By promoting clear communication, empowering patients, and developing comprehensive support structures, healthcare providers can strengthen patient-centered care pathways and enhance both quality of life and care experiences for this vulnerable population. Ongoing efforts to explore these dimensions are essential for optimizing care practices and outcomes for patients living with brain metastases.

Brain metastases (BM) are the most common intracranial neoplasm in adults, affecting 20%-40% of cancer patients,^1^ and frequently arising from lung, breast, colorectal, renal, and melanoma tumors.^2^ The incidence is rising, driven by increasing cancer prevalence, improved survival given therapeutic advances, and enhanced and more frequent neuroimaging techniques.^1^,^3^,^4^ Median survival remains poor, generally ranging from 3 months to 1 year.^5^

Brain metastases impose substantial physical and cognitive burdens that affect daily functioning and quality of life (QoL) for both patients and caregivers.^6^ Common symptoms include headache, nausea and vomiting, seizures, focal neurological deficits, cognitive or mental changes, and fatigue.^6–8^ Despite this, few prospective studies examined how BM influence daily life.^9^ A systematic review^10^ of information and supportive care needs in patients reported poorer QoL among BM patients compared to those with other advanced cancers. Existing research has largely focused on physical needs, prognosis, and treatment options. Consequently, studies exploring how patients with BMs experience daily life, including their functioning, concerns, and worries, remain limited.

Previous qualitative research has primarily addressed malignant brain tumors. A recent systematic review^11^ synthesizing 33 studies identified profound unmet needs for tailored information, care coordination, emotional support, and the value of holistic needs assessments and early palliative care. However, patients with BM were explicitly excluded from the study, leaving a critical evidence gap. BM patients represent a large and growing population with distinct clinical trajectories, shorter prognosis, and unique informational and psychosocial challenges compared to those with primary brain tumors. These distinctions are reflected in a recently published consensus-based framework introducing 29 quality indicators for multidisciplinary BM care.^12^ The framework emphasizes patient-centered measures such as information quality, shared decision-making, early symptoms, and QoL screening, and psychosocial support. At the same time, the expert panel identifies an implementation gap in patient-centered care (PCC), underscoring the need for in-depth, longitudinal patient perspectives to better inform and guide practice.

A PCC approach is essential for individuals with BM, as it prioritizes personal needs, preferences, and values, including treatment decisions and care provision.^13^ PCC is a cornerstone of high-quality care and widely promoted to improve practice. Within this model, patients are viewed as active participants supported to take ownership of their health and well-being.^14^ To address the evidence gap in patients' experiences of being diagnosed with and living with BMs, and to inform PCC, we conducted a qualitative study employing semi-structured interviews with patients recently diagnosed with BM over the first 4 months post-diagnosis. Our research questions were interrelated and addressed different aspects of this overarching aim. The first question served as the primary analytic focus, while the others provided complementary insights into care experiences and daily life:

- How do patients experience being diagnosed with BM?
- How do they perceive the care they receive, including the provision of information and involvement in treatment decisions?
- How do BM affect their daily lives, and what are their primary concerns?

## Methods

This study was part of the research program *Brain Metastases in Norway* (2013-2016*),*^15–18^ which aimed to characterize treatment, outcomes, and symptom burden in patients with BM while identifying potential improvements in care delivery. Patients with a first-time BM were recruited from 3 hospitals within Oslo University Hospital catchment area in south-east Norway.

Inclusion criteria:

- Confirmed diagnosis of a solid tumor
- BM verified by computed tomography, contrast-enhanced magnetic resonance imaging, or biopsy
- Age ≥18 years, physically and cognitively able to participate
- Provided written informed consent
- Willingness to attend 2 or 3 interviews

Exclusion criteria:

- Primary brain tumor
- Prior diagnosis/treatment of BM
- Cognitive, mental, or physical impairment precluding study compliance
- Inability to provide written informed consent in Norwegian

Participants were recruited by an oncologist and a study nurse during initial hospital visit after BM diagnosis. Using purposive sampling, recruiters selected patients representing the target population with variation in age, sex, and diagnosis. The recruitment target was 20 participants to ensure data saturation.

### Design, Setting, and Data Collection

A longitudinal design with interviews at 1-3 weeks (I1), 2 months (I2), and 4 months (I3) post-diagnosis was employed to capture evolving symptoms, functional decline, and care needs, using short intervals due to limited life expectancy.

Interviews were conducted during hospital visits, except for one by phone due to COVID-19. Two trained cancer nurses (Wester [female] and Hansen [male]) led the interviews, with the first done jointly for consistency. Patients were informed that interviewers were not part of the care team, had no access to medical records, and responses would not affect treatment. In 2 instances, consent was obtained to alert physicians about untreated symptoms.

Following each session, participants reflected on the interviews, but no significant concerns were reported. The interviews followed a structured interview guide developed and piloted by the research team (Table 1). The first interview focused on the BM diagnosis and the content of the initial consultation, including involvement in treatment decisions. All interviews addressed everyday life, symptoms, functioning, worries, and priorities, with particular attention to what mattered the most and unmet information needs. Participants were invited to share additional thoughts or questions. This approach addressed study aims while enabling free comments.

**Table 1.** Interview guide.

Theme	Example questions
Experience of diagnosis	Can you describe what it was like when you received the diagnosis of brain metastases? How did you first learn about the diagnosis?
Consultation & decision-making	What did you and your doctor discuss at the time of diagnosis? How were you involved in decisions about your treatment? Were different treatment options explained to you? How?
Everyday life	How have you managed daily life since the diagnosis? Have you noticed any changes in your physical, social, or emotional functioning?
Worries and priorities	What concerns you most right now? What is most important to you at this stage? Do you feel you need more information or support?
Additional reflections	Is there anything else you would like to share that we haven’t discussed? How has it been for you to participate in this interview?

Field notes were taken, and interviewers debriefed after each session. Interviews lasted from 16 to 58 minutes (mean: 32 minutes), were audio-recorded, and transcribed verbatim by professionals. No personal identification data were recorded. For the purposes of preparing the manuscript, illustrative quotes were translated into English. Translations were done by the first authors, then cross-checked by co-authors and a proofreading service.

### Methodological Orientation and Analysis

A qualitative, exploratory, longitudinal design^19^ grounded in an interpretivist orientation emphasizing PCC and subjective experiences in the context of serious illness.^13^,^14^ Data were inductively analyzed using Bingham’s 5-phase process,^20^ allowing themes to emerge from the data and ensuring transparency.

The analysis involved familiarization with the data, coding, theme identification, relevance review, and integration with existing literature. Two of the authors, T.L. (researcher with background in psychology and behavioral science) and T.E.W. (cancer nurse with master’s degree), conducted the initial analyses. They first read all interviews to get a broad understanding, then inductively coded the baseline interviews by hand to identify common topics. An iterative coding tree organized codes and topics, yielding 7 for longitudinal analysis (Table 2) based on frequency, salience across participants, and relevance to the research questions. Using longitudinal trajectory analysis,^21^ a matrix was created in Microsoft Excel for each patient to explore changes over time across the 7 topics. The complete set of matrices was then reviewed, resulting in 4 overarching final themes. These were discussed and refined through consensus in an extended group. All group members read interviews, and agreement was reached after discussion. Data sufficiency was assessed continuously, and saturation deemed reached when no new codes or themes emerged. Despite attrition, thematic saturation was achieved for the primary research questions.

**Table 2.** Characteristics of the participants.

Patient number	Sex	Age	Diagnosis	Treatment	Educational level	Number of interviews
1	F	44	Breast	Surgery	University >4 y^a^	3
2	F	73	Lung	SRT^b^	University <4 y^a^	3
3	M	73	Lung	Surgery	High school	3
4	F	65	Lung	WBRT^c^	University >4 y^a^	1
5	F	61	Breast	Surgery	University >4 y^a^	3
6	F	81	Lung	WBRT^c^	Unknown	1
7	F	59	Lung	WBRT^c^	University >4 y^a^	3
8	F	55	Breast	SRT^b^	University >4 y^a^	3
9	M	81	Gastrointestinal	Unknown	High school	2
10	F	61	Lung	WBRT^c^	University >4 y^a^	1
11	F	62	Breast	Surgery	Unknown	3
12	F	79	Gastrointestinal	WBRT^c^	Elementary	1
13	F	62	Breast	WBRT^c^	High school	2
14	M	78	Gastrointestinal	SRT^b^	University >4 y^a^	2
15	M	60	Gastrointestinal	Surgery	High school	1
16	M	81	Melanoma	WBRT^c^	University <4 y^a^	1
17	M	65	Melanoma	SRT^b^	High school	3
18	M	43	Melanoma	WBRT^c^	University <4 y^a^	1
19	F	77	Melanoma	Systemic	High school	3
20	M	79	Melanoma	SRT^b^	University <4 y^a^	1

F = female, M = male.

a Years.

b Stereotactic radiotherapy.

c Whole brain radiotherapy.

### Reflexity

Researcher bias was acknowledged as a potential influence on data collection and interpretation.^22^ The interviewers (T.E.W. and S.B.H.), experienced cancer nurses outside the treatment team, clarified their roles to foster trust and openness, though interaction dynamics may have shaped responses. Primary analysis was led by T.E.W. and T.L. whose backgrounds likely influenced interpretations. To mitigate bias, interpretations were challenged and discussed within the extended research group, which included an oncologist (O.E.Y.), the project leader (N.A.), a user representative, and a researcher with broad knowledge of qualitative analyses (A.B.).

### Ethical Approval

The study adhered to the Helsinki Declaration and complied with the General Data Protection Regulation. The Regional Committee for Medical and Health Research Ethics, South-East Norway (REK reference: 2018/800), as well as the Hospital Data Protection Officer approved the study. Written informed consent was obtained from all participants. All data were de-identified, securely stored on a password-protected server according to institutional policies.

## Results

The study (April 2019-April 2021) enrolled 22 patients. One patient withdrew before I1 and another did not complete I1 due to psychological strain, leaving 6 patients with lung cancer, 5 with breast cancer, 5 with melanoma, and 4 with gastrointestinal cancers (Table 1). Median time from primary cancer diagnosis to first BM was 30 months (range 0-183). The median interval was 7 months (range 0-64) between extracranial metastases and BM, while 3 patients had no prior extracranial spread. Eight patients either died or were too ill to participate in I2, and 3 patients could not complete I3 for the same reasons. In total, 41 interviews were conducted lasting 16 to 58 minutes (mean 32). Of 20 participants completing I1, 9 completed all three interviews, 3 completed two, and 8 completed one. Women (7 of 12) were more likely than men (2 of 8) to complete all 3 interviews.

The analyses identified 4 overarching themes: (1) *Brain metastases*; Either an additional burden or more of the same, (2) *Trust*; Patients' confidence in the health-care system, despite limited involvement and substantial information needs, (3) *Distancing*; Despite bleak prospects, illness and death are kept at arm’s length, and (4) *Acceptance and adjustment*; Adapting to a considerable symptom burden and impaired activities of daily life.

### 1. Brain metastases; Either an additional burden or more of the same

Participants' reactions to being diagnosed with BM fell into 3 categories: relief, acceptance, or distress. Those who had endured unexplained symptoms like severe headaches, nausea, or vomiting over time, finally got an explanation offering prospects for symptom control. One patient said:

- *It was perhaps more of a relief at first, because I hadn’t realized what was wrong with me, and for a long time I had been very strange and dizzy, and clumsy … had lots of strange lights that I saw, I thought it was stroke.* (Patient (P) 8, I1)

Other patients said they acknowledged the progression as a part of the disease.

*- It’s a message that you must take, which I took on board, and probably just thought that here we’ll just have to follow the race and then we’ll simply see what happens.* (P22, I1)

There were patients with more emotional reactions:

*- She says, you have cancer in the lungs, which has spread to the skeleton and up to the brain. And then I thought I was going to die, quite simply. Because it was so - (Crying) so shocking, and so far beyond anything I had imagined it could be. So, really, I just shouted—That’s it, you just roll around in panic and in pain and everything. It’s like that, yes, it’s like falling and falling, falling, falling and then there’s no one to pick you up.* (P4, I1)

While many patients expressed feelings of distress, sadness, disappointment, fear, shock, and disbelief, only a few expressed explicit concerns about the brain being the location of the metastases. Some asked about the reasons and timing of their cancer’s spread to the brain, why previous treatment had not prevented further progression, and whether the metastases would recur. Few patients raised concerns about being cognitively affected in the future. Three patients described short temper. Some mentioned forgetfulness but did not find it particularly worrisome:

*- So, that’s what worries me a little, because it has been suggested that it might have come from that colon, then I think—Why does it do that? I was supposed to be cancer free. Why haven’t they followed up on me?* (P10, I1)

Theme 1 primarily originated from the responses during the first interview. However, the longitudinal analyses showed that patients brought up inquiries about why the cancer had spread to the brain more frequently in I2 and I3. Practical challenges like losing a driving license and increased dependence on others were more common in later interviews. Patients were overall satisfied with how the diagnostic information was conveyed, especially when being informed by a physician they had met before. They emphasized the importance of not being alone when receiving bad news, both for emotional support and to help remember the information provided. One said:

*- I would have preferred to have someone with me. When I get these heavy messages, it really helps to have someone to, well, to cry with, or to receive the same information, because that’s at least the experience I have, that I don’t always catch everything when I’m in those doctor’s appointments, and then my husband acts a bit like a secretary. Then we can discuss the information afterwards, and he often takes some notes…* (P1, I1)

### 2. Trust; Patients' confidence in the health-care system, despite limited involvement and substantial information needs

Overall, patients felt confident that they would receive optimal BM treatment. When questioned about involvement in treatment decisions, they reported receiving a plan outlining the treatment type and schedule. None mentioned having discussions with the responsible physician or the care team about the plan and potential alternatives for treatment. Most participants had fully trusted their physicians' decisions, as illustrated with these citations:

- *I trust that they can fix me*. (P3, I1)

- *There was no reason not to follow that advise*. (P16, I1)

*- We have different skills in life. My expertise extends to many other areas, but not when it comes to this*. (P22, I1)

Overall, the patients seemed satisfied with this approach. They expressed more concern regarding the effectiveness of treatment, rather than its appropriateness. Many praised the health care system and expressed a profound gratitude:

*- Ever since I got here, everything has gone smoothly, nothing to complain about really*. (P8, I1)

However, there were some exceptions. A few patients were dissatisfied and wanted more involvement in treatment decisions. They reported an excessive focus on anti-cancer treatment, felt expected to accept treatment, and were afraid that further care would be compromised if they declined. When the interviewers asked if the patient had expressed doubts about more chemotherapy to the physician, one patient said (P14, I1):

*- P: No, I didn’t say anything, no.*

*- I: No…?*

*- P: Because I thought.., because I’ve experienced that, that, doctors often cope very poorly when I make the decisions for myself.*

Patients' evaluation of information ranged from high satisfaction to feeling uninformed, illustrated by these statements:

*- The information suits me. I’m an old businessman, so I think it’s fine. Matter of fact, clear, no speculations one way or the other—just this is how it is now. So that’s fine*. (P22, I1)

*- It feels as if nobody tells me anything*. (P12, I3)

*- … Also, I do not get the information I need to be able to make my own choices/decisions*. (P14, I2)

Analysis revealed that many patients had a limited understanding of their illness, treatment, or prognosis. Some even reported receiving no information about the intent of their treatment. Several patients wanted more details on treatment side effects and long-term implications, expressing concern about unforeseen consequences. One noted:

*- Beyond that (a little dizziness), I wasn’t told that it (brain radiotherapy) would have any consequences. If something happens in the long term, no one has mentioned it. It is interesting to think about what happens moving forward. Are there any long-term consequences that I’m not aware of? (…) What can I expect…what is the range of possibilities?* (P5, I3)

Some patients had forgotten, misunderstood, or not received details about their diagnosis. For instance, one repeatedly shared information suggesting misinterpretations during the initial interview:

*- And when I was told that there was also something in my head, I was (…), I thought I can’t do anything about that either. And then they said it was dots. They didn’t say tumor. And I thought; maybe it’s something that can be treated then*. (P21, I1)

Others stated clearly that they didn’t want to know or even think about what might come. In the longitudinal analyses we observed changes over time; some patients increased their knowledge about diagnosis and treatment, while others became increasingly aware of gaps in their understanding. This growing awareness led them to ask more treatment-related questions and seek additional information.

### 3. Distancing; Despite bleak prospects, illness and death are kept at arm’s length

Across all interview time points, most participants focused on maintaining normalcy in daily life, prioritizing work, family activities, and ordinary conversations over discussions about cancer, treatment, or the future. Several informants resisted talking about the disease, valuing activities that preserved their identity and sense of self. Maintaining their identity was important, having normal days, doing normal things. By focusing on the disease, some felt they lost themselves. One said:

*- It is important to me that I am me, not only the disease*. (P1, I1)

Many participants focused on taking one day at a time. They prioritized family and daily life. None had discussed future needs with their care team and focused on improvement and “getting well.” Many said they chose to stay positive and avoid dwelling on the cancer. They described themselves as optimistic persons and expressed a belief in a positive outcome:

*- We’re not worried about the outcome; I do not believe this isn’t going well*. (P5, I1)

When asked about hope, one patient expressed it this way:

*- That I have several relatively normal years to come, and plenty of time with my family—That’s what I’m hoping for… and that it doesn’t get too tiring with too much chemo (many chemotherapy treatments) and things like that…* (P9, I1)

Whilst participants seldom voiced worries about the future, they sometimes had utterances indicating that this was on their mind. Several patients said that their physician had assured that everything would be fine. Others expressed concern about treatment effect:

*- I was well informed about my prospects, they (health care personnel (HCP)) were confident that things would go well, and then I calmed down*. (P19, I1)

*- Whether I’m never going to be well, I guess that’s what worries me the most. I have hope that everything will be fine and that I’ll be normal again. One must believe that*. (P19, I2)

Few participants openly expressed thoughts about death and dying, and when doing so it mostly concerned a reduced life expectancy. These expressions were often in a positive outlook, underlining that they were doing fine after all.

*- Many strange thoughts come to mind when thinking about death and stuff (…) many strange thoughts. But I think we should…- I think it’s going well*. (P12, I1)

### 4. Acceptance and adjustment; Adapting to considerable symptom burden and impaired daily life activities

While patients focused on trying to keep normalcy in daily life, many had adjusted their activities substantially due to symptom burden and impaired functions. This was not explicitly described but became evident during the analyses. Patients often described their performance status as good and said that they were doing well, living like usual. Yet, the participants' perception of a “normal” life had changed. During the interviews it became obvious that their social life was limited, their functions impaired and that physical activity had been substantially reduced. Only a few were able to work. Headaches, tiredness, fatigue, dyspnea, and dizziness were frequent, but none described any cognitive decline. Despite a high symptom burden patients described their condition as acceptable:

*- I’m actually doing ok, it’s just when I stand up…, I can no longer go for walks, and I cannot stand while cooking.* (P12, I2)

Whilst some responded well to treatment and improved, the condition of others declined due to disease progression or side effects of treatment. Overall, patients expressed a great adjustment to their situation and seemed to have lowered their expectations of what everyday life should entail. Some patients described creating a new normal, framing it as a cognitive process:

*- I believe that over time the tension level, let’s say, gradually decreases more and more, and you transition into a situation where you feel that this is a kind of everyday life that we live in and which simply… is the day, it’s our everyday life. And in a way, that’s okay*. (P22, I1)

During the interviews, the acceptance of the new normal increased. One of the patients described accepting and adapting more over time:

*- It’s all about figuring out, what I call “the new normal,” and that takes time (…), because I do not longer know what normal is, or what it entails.* (P8, I2)

## Discussion

This study provides new insight by examining in depth how patients newly diagnosed with BM perceive and experience their situation. In a field with limited qualitative, in‑depth research, our findings may help improve and better tailor the care provided to this patient group. The patients reported varied emotional responses to their BM diagnosis, ranging from relief to distress. Their experiences highlight the importance of information and involvement in decisions. Our findings underline that PCC must go beyond symptom management to address broader dimensions of care.

Most patients did not express specific concern about the brain being the site of metastases or fears related to cognitive decline or shortened lifespan. It is unclear whether this reflects limited understanding, lack of information, or an intentional avoidance of difficult topics. Insufficient tailored information may also have contributed since several patients seemed to accept their situation partly because they did not fully grasp its implications. Over time, however, they began asking more treatment-related questions and expressed an increasing need for additional information. For some patients, especially those with unexplained symptoms, the diagnosis appeared to bring relief by resolving uncertainty and enabling treatment. This relief was often coupled with distress over disease progression, future implications, and fear of death, though few raised these issues directly. Instead, these hidden concerns surfaced subtly throughout the interviews and seemed to shape many emotional responses.

These findings align with other studies showing mixed emotional responses of BM patients.^23–25^ Coexistence of relief and distress is common and may reflect the struggle to adjust to a life-threatening condition.^25^ Focusing on normalcy and distancing themselves from the diagnosis may function as coping mechanisms.^26^  This underscores the need for HCPs to anticipate varied responses and tailored communication. Clear, empathetic communication, repeated conversations, psychological support, and caregiver involvement may strengthen PCC.^23^^,^^27^

Patient experiences with information varied widely. Some expressed increasing need for details about side effects, prognosis, and long-term consequences, while others misunderstood, forgot, or lacked key information. Several were unaware whether treatment was curative or palliative. In contrast, a few preferred not to know. These variations underscore the importance of individualized communication,^23^ and highlight the need to understand the patients' concerns and needs over time.

During the first interview, many patients seemed to prioritize information related to their immediate condition, such as symptom management, rather than what’s ahead. This contrasts slightly with findings from studies on patients with primary brain tumors, who actively sought information, emphasizing that sufficient information empowered them.^28^ A systematic review of 22 studies involving patients with various cancers found that nearly all patients desired comprehensive information about prognosis, diagnosis, and treatment options.^29^ Interestingly, the patients with BM in the current study seemed less concerned about this. A possible explanation could be that many of these patients already had cancer prior to the BM diagnosis, and their symptoms were finally explained by the discovery of the metastases. This contrasts with patients diagnosed with a primary brain tumor, where the diagnosis itself often comes as a first and unexpected situation. Awareness of a diagnosis linked to limited life expectancy may have contributed to a stronger emphasis on managing immediate concerns rather than prospects. However, our analyses showed that patients' informational needs shifted from symptom management to understanding why their cancer spread to the brain and its broader implications, underlining the values of repeated assessments.

While respecting patients' initial desire to avoid detailed information, it is crucial to ensure they are adequately informed. Individualized information, an essential part of PCC, ensures that each patient’s unique needs, preferences, and values are considered and can help alleviate concerns.^23^,^24^,^27^ HCPs should also ensure that the patients comprehend and understand the implications of their diagnosis particularly because the understanding of information among patients with BM could be compromised by cognitive impairment.^27^ Such practices are recognized quality indicators of PCC for patients with BM.^12^

Contrary to our initial belief, patients were generally not interested in being directly involved in treatment decisions. Their trust in their medical team appeared positive for their emotional wellbeing, as observed in a previous study on BM patients, finding that trust and a sense of hope were key factors in improving emotional outcomes.^23^ Nevertheless, trust was not universal. For some patients who desired greater involvement, the perception that treatment options were presented as nonnegotiable led to feelings of dissatisfaction, highlighting the importance of balancing trust with opportunities for involvement. It is also possible that some patients avoided involvement and did not seek additional information because they believed they would recover soon, as suggested in another study on BM patients.^28^ Recognizing the difference between trust and denial is critical, as denial may lead to challenges for both patients and caregivers. Since preferences for involvement vary widely, HCPs are advised to regularly assess each patient’s desired level of involvement in treatment decisions.^29^ Although many patients in our study preferred to delegate decisions, they expected clear information and the opportunity to revisit preferences over time. Systematic preference elicitation and informed consent rather than a one-size-fits-all approach are essential.^12^

The patients reported that the BM diagnosis profoundly affected their lives, forcing them to adapt to a new normal. Although they tried to adjust to their new reality, the diagnosis brought significant challenges. This finding aligns with Nyatanga et al,^30^ who reported that brain tumor patients felt the need to adjust their lives and ways of thinking to cope with their condition. Similarly, a Norwegian study^31^ found that older adults with incurable cancer prioritized living as long and as well as possible, emphasizing adaptation and meaning-making as central to coping and maintaining QoL. Despite efforts to maintain normalcy, our findings showed that BM significantly impacted patients' daily lives. Participants often understated or normalized frequent symptoms, describing their condition as “acceptable.” This is illustrated by the following quotation, “I’m actually doing ok, it’s just when I stand up, and that I can no longer go for walks….” Since patients may not voluntarily disclose symptoms, clinicians should actively explore and address symptoms and functioning during consultations. Regular follow-ups with patient-reported outcomes are a prerequisite for individual follow-up. Reduction in physical activity, limited social lives, and inability to continue working point to substantial life changes and likely reflect lowered expectations for daily life. While some patients expressed gradual acceptance and even a sense of peace with their circumstances, others struggled to reconcile their pre-diagnosis lives with their current limitations.

A focus on maintaining normalcy and adapting to challenges may reflect resilience.^32^ The ability to adjust to the physical symptoms, social limitations, and functional changes likely protects them from psychological distress and helps maintain emotional balance. The literature shows that resilience is shaped by biological, personal, and social factors such as optimism, hope, and support from family, friends, and healthcare providers.^32^ This underscores the need for interventions that strengthen resilience and support patients in managing the psychological and existential challenges of living with BM.

### Strengths and Limitations

A strength of this study was the use of a semi-structured interview guide organized around 5 predefined themes, which ensured consistency while allowing flexibility. The open-ended format enabled participants to express in their own words and raise issues beyond the planned topics, making it possible to capture unexpected findings such as the emphasis on maintaining everyday life. The PCC approach enhanced the validity of the data. Another strength was the longitudinal design. Conducting interviews at multiple timepoints allowed for observation of changes and development in patients' perspectives and may have contributed to trust and more open communication across interviews.

The patients' vulnerable situations and poor health represent limitations of this study. Although we excluded the most severely ill and used short intervals between interviews to maximize participation, attrition was high and fewer than half completed the full series. High dropout rates are common in longitudinal studies involving patients with advanced illness,^33^ often reflecting deteriorating health. However, this may introduce bias and limit the ability to capture complete longitudinal trajectories, as it reduces data richness, narrows the diversity of perspectives, and hinders the continuity needed to follow changes over time. Consequently, the findings may overrepresent the experiences of participants with better health or resilience. Furthermore, women were more likely than men to complete all 3 interviews, meaning that the later data primarily reflects female perspectives. This imbalance may influence themes such as coping, information needs, and adaptation, and may limit transferability to male patients. Together, these factors underscore the need for cautious interpretation of the findings and highlight the importance of strategies to minimize dropout in future studies, as well as the value of considering gender differences in research design and analysis.

When developing the interview guide, we assumed that a BM diagnosis would be perceived as significantly worse than other serious cancer-related news, which carried a risk of bias. However, the interviewers were aware of this risk and were careful to avoid leading questions. No evidence of such bias was identified during the review of the interviews.

## Conclusion

In conclusion, patients' experiences of being diagnosed with BM are multifaceted, encompassing a range of emotional reactions, practical concerns, and questions about disease progression. Although many accept and adjust to new normal, impaired functioning, inadequate symptom control, and unmet information needs may impair their QoL. Patients were generally satisfied with their care and felt confident in receiving the best treatment, with most preferring to leave decisions to their physicians. These findings highlight the importance of a patient-centered approach emphasizing clear communication, proactive symptom management, and tailored care to better support the patients. Further research should explore strategies to address their unmet needs. Implications for clinical practice are presented in Table 3.

**Table 3.** Specific recommendations for clinical practice.

Key area	Actionable steps
Tailored information provision	Regularly assess and revisit patients' understanding of their diagnosis, treatment options, and prognosis. Use clear and empathetic communication and involve family members or caregivers during critical discussions.
Proactive symptom management	Implement routine symptom assessment tools to identify and address pain, cognitive changes, and emotional distress. Encourage open dialogue about symptoms and their impact on daily life.
Patient involvement in treatment decisions	Foster a collaborative decision-making process by regularly discussing treatment plans and alternatives. Ensure that patients feel empowered to voice their preferences and concerns.
Addressing unmet information needs	Develop comprehensive support systems that address common patient questions about prognosis, treatment side effects, and long-term implications. Provide opportunities for follow-up questions and discussions.

## Data Availability

The data supporting the findings of this study are not available in a public repository; however, the data may be made available upon reasonable request and with participants' consent, upon request from the first author, Asta Bye (abye@oslomet.no). The data are not publicly available due to ethical considerations, including the need for anonymization and the protection of participants' confidentiality.
